# Preliminary Characterization of Novel LDPE-Based Wear-Resistant Composite Suitable for FDM 3D Printing

**DOI:** 10.3390/ma12162520

**Published:** 2019-08-08

**Authors:** Piotr Olesik, Marcin Godzierz, Mateusz Kozioł

**Affiliations:** Faculty of Materials Engineering and Metallurgy, Silesian University of Technology, Krasinskiego 8 Street, 40-019 Katowice, Poland

**Keywords:** FDM printing, glass wastes, low-density polyethylene, composite, friction, wear

## Abstract

Low-density polyethylene (LDPE) composites reinforced with finely powdered waste glass were identified as a potential material for 3D printed structures for use in low-duty frictional applications. A recently published 3D printing model was used to calculate the limits in the filament feed rate and printing speed. Tribological tests (pin-on-disc method) of the printed composites were performed for different print-path directions. Differential scanning calorimetry (DSC) was performed on the samples and the composites showed a higher crystallinity compared with LDPE, which partially explains the higher elastic modulus of the composites determined during static tensile tests. Using a fine glass powder as reinforcement improved the wear resistance of LDPE by 50% due to the formation of a sliding film on the sample’s surface. An evident effect of friction direction vs. the printed path direction on wear was found; which was likely related to differences in the removal of friction products from the friction area for different print-path directions. The LDPE composites with fine waste glass particles are promising materials for low-duty frictional applications and should be the subject of further research.

## 1. Introduction

Additive manufacturing (AM) is a novel and revolutionary technique where it is possible to produce elements with complex geometries directly from the desired material [1]. For thermoplastic materials, the most common AM technique is fusion deposition modeling (FDM) because it is very simple, relatively cheap, and is a kind of 3D printing [2] that can be fully automated, which is becoming increasingly common in materials technologies [3]. One of the disadvantages of using FDM is the low mechanical strength of the products, which can only be produced from some types of thermoplastics, which limits the usage of FDM technology [4]. A possible solution is the application of strong materials, such as polyetheretherketone (PEEK), but such materials are very expensive and not readily available. An alternative method is the use of thermoplastic composites reinforced with hard particles or short fibers. Such FDM printed composites show much better properties, such as higher strength, improved wear resistance, and better dielectric permittivity compared with neat thermoplastics [5,6]. The addition of reinforcement materials can also help to solve some common problems with the 3D printing of neat polymers. For example, adding iron or copper particles to ABS reduces the distortion of printed parts [7,8]. Currently, composites reinforced with short fibers, long fibers, powders, or nanocomponents can be found in many solutions [9,10,11] and are also applicable for 3D printing [4,12].

**Figure 1:**
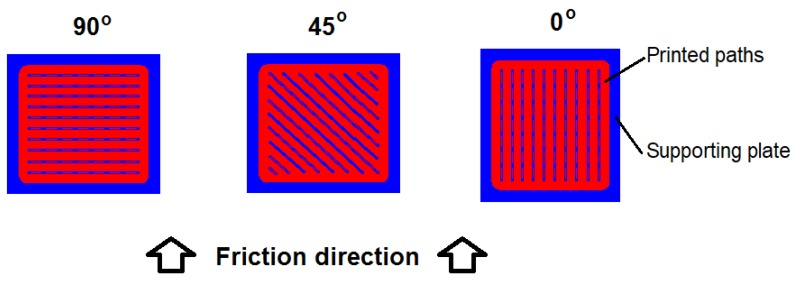
3D printout with possible anisotropy vs. friction direction.

The wear behavior of 3D printed composites is a relatively new topic that has mainly been researched for ABS or PC-ABS blends [13,14]. The wear resistance of Nylon-6 composites has also been described [15]. Since 3D printed parts exhibit anisotropic mechanical properties [16], their wear mechanism may also vary depending on the friction direction (Figure 1). Thus, such behavior should be more thoroughly investigated.

LDPE is an inexpensive and very popular material that is currently used mainly in packaging, toys, and everyday-use items, which generate significant waste that must be recycled. The reuse of polymer wastes as filaments for FDM printing is a relatively new and popular topic; there are works [15] describing the use of polymer waste to obtain material for 3D printing. However, LDPE has a relatively low strength and stiffness, poor bed adhesion, and high shrinkage, which may disqualify it from being used in FDM printing. Nevertheless, some problems can be solved by reinforcing LDPE with hard particles to produce composites [9,17]. As mentioned before, adding ceramic or metal powders to LDPE can improve its storage modulus [8], reduce shrinkage, and increase its mechanical properties [7]. Currently, LDPE composites with a mold flow index (MFI) of 10 g/10 min were successfully printed [18], so it is possible to manufacture an LDPE composite filament for FDM printing made from waste materials.

The use of LDPE as a friction material is not common, but it can be used in new applications where frictional properties and wear resistance are of great importance. An example of this is the mesosterephs in the soles of shoes, where in addition to the necessary friction properties, a low hardness, plasticity, and elasticity are important. It may also be a good carrying material for solid lubricants [19], e.g., as a layer or near-surface filler. Other mass applications include novel high-endurance sliding pads for furniture, such as chairs and stools. In the rapidly innovating field of consumer products, many other applications for thermoplastics (e.g., [20]) and FDM technology can be imagined [21].

Mathematical descriptions of FDM 3D printing are still being investigated. The problem of control and performance limitations can be considered in relation to melting in a liquifier or layer deposition [22,23,24,25,26,27,28]. Recent publications describing this issue have shown that mathematical modeling can be used to estimate process parameters, and to also avoid potential 3D printing failures, such as buckling or filament grinding. For example, Mackay et al. [26] attempted to determine the minimum required temperature of feed filaments. The experimental evaluation and mathematical prediction of FFF limits were performed by Go et al. [25] and Turner et al. [23] previously provided a review of melt extrusion descriptions. Osswald et al. [24] proposed a model based on the formation of a thin melt polymer film in a nozzle, which made it possible to estimate the maximum filament feed rate in which the melted polymer is pushed through a nozzle by a fed filament. The force applied by the filament was proportional to its feed rate. When the filament moves faster than its tip is melted, failure from buckling or grinding can occur. One way to prevent this is by matching the filament velocity to the feed rate. The maximum feed rate can be calculated using the least-squares method if the tensions in the filament are equal to the force necessary to push melt through the nozzle.

The aim of this study is to characterize a new composite suitable for FDM printing, which is inexpensive—making it suitable for mass production—and has enhanced wear resistance. In previous work, the authors made an LDPE composite reinforced with glass waste (an additional eco-role) as a 3D printing material [29] with a melt flow index (MFI) of 2 g/10 min. Glass powder obtained from shredded car windscreen wastes was used as a reinforcement material to increase the printability of LDPE. In this work, the authors continue the attempts to characterize this material in two areas: its technological properties and wear resistance. A previously described mathematical model was applied to establish the buckling conditions and to estimate the maximum feed rate of the filament. Tribology tests were performed on 3D printed parts to determine whether glass wastes can be used as a reinforcement material to enhance the wear resistance of 3D printed parts and if the friction direction influences the specific wear rate of 3D printed composites.

## 2. Materials and Methods

**Figure 2:**
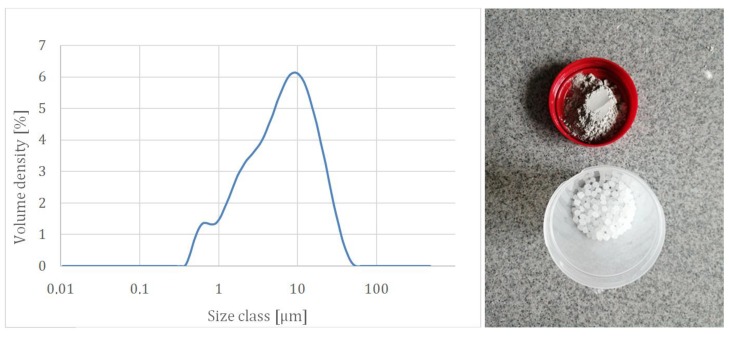
The particle size distribution of the obtained glass powder (left) and the powder with low-density polyethylene (LDPE) granules (right).

The matrix material was LDPE (Malen E FGNX 23-D022, Basell Orlen Polyolefins, Płock, Poland) with an MFI (190 °C/2.16 kg) of 1.95 g/10 min. The reinforcement material was glass powder containing polyvinyl butyral (PVB) obtained from laminated car glass waste. In this work, composites with 15 and 30 vol% of powder were made, along with neat LDPE as the reference. The glass pieces were mechanically cleaned from the outer layers of PVB foil and initially milled in a hammer grinder mill Retsch SK300 (Retsch, Haan, Germany) with a sieve mesh diameter of 0.5 mm. To obtain a fine powder, a planetary ball mill (Fritsch Pullverisser 6) was used. During milling, the powder-to-ball ratio was 1:4, and the milling speed was 600 rpm. An effective milling time of 60 min resulted in a powder of D90 = 20.1 μm with no grains larger than 100 μm (Figure 2).

Gradual mixing is a common technique to achieve powders with a wide range of particle diameters [10,30]. The obtained range of particle diameters was the minimum one expected to ensure the efficiency of the FDM printing process and at the same time the smallest effect on the cost of the material.

The obtained powder was mechanically mixed with LDPE granules, dried at 70 °C for 24 h, and then extruded. Obtaining the glass used to produce the powder is relatively simple and cheap, especially compared with advanced methods such as those described in [31] or [32]. It is rather more similar to more massive technological methods applied for modern materials [33,34].

After the first extrusion using a ZAMAK DTR EHP-2x16S (Zamak Mercator, Skawina, Poland) extruder, composites were re-granulated and extruded again to obtain a homogenous material, which was extruded in the form of a filament with a diameter of 1.45 ± 0.10 mm that was suitable for 3D printing. The nozzle temperature during extrusion was 190 °C, screw speed 28 rpm and winder speed 8 rpm.

The microstructure of the obtained filament and the distribution of glass particles in the polymer matrix were evaluated using a Hitachi S-3400N (Hitachi, Tokio, Japan) scanning electron microscope (SEM).

Differential scanning calorimetry NETZSCH DSC 404 F1 (Erich Netzsch GmbH, Wunsiedel, Germany) was used to determine the enthalpy of fusion to calculate the critical filament feed rate. The measurements were carried out in argon (gas flow 20 mL/min) and with heating/cooling rate 5 K/min. Along with this, the crystallinity of neat LDPE and the composites was determined using Equation (1) [35,36,37,38]:

(1) $$\chi \%=\frac{1}{1-\Delta wt\%}\frac{\Delta H_{c}}{\Delta H_{f}^{0}}$$

where wt% is the mass percentage of the reinforcement, ΔH_c_ is the crystallization enthalpy [J/g], and ΔH_f_^0^ is the theoretical crystallization enthalpy of 100% crystalline LDPE [293 J/g].

To confirm crystallinity results from DSC analysis, x-ray diffraction (XRD) tests of the neat LDPE and the composites were performed. The measurements were taken on bulk samples at Panalytical Empyreal at room temperature using Cu kα radiation (λ = 1.5405 Å), scanning step of 0.03° from 10° to 80° of 2Ɵ (Bragg angle) and 45 kV/40mA. In order to obtain reliable results, XRD curves were normalized. The crystallinity was calculated with Equation (2):

(2) $$\chi \%=\frac{A_{C}}{\left(A_{C}+A_{A}\right)}\times 100\%$$

where: A_c_—the area of the crystal region, A_A_—the area of the amorphous region, A_c_ + A_A_—total area.

In a previous publication [29], the produced filaments were subjected to a static tensile test. The length of the measuring base was 100 mm, the deformation speed was 25 mm/min, and the device used was an INSTRON 4469 (Instron, Norwood, MA, USA). The results from this test were used to obtain the Young’s Modulus of all materials, and the calculated values are listed in Table 1 and were used in further calculations.

The power-law relation for the all obtained composites was approximated from viscosity vs. shear rate curves. The values of 〈γ〉 and τ were obtained from MFI measurements performed on CEAST plastometer (Instron, Norwood, MA, USA) using the following Equations. (3) and (4):

(3) $$\tau =\frac{R_{N}F}{2\pi R_{p}^{2}l}$$

where *R*_N_ is the nozzle radius [0.9 mm], *R*_p_ is the piston radius [4.6 mm], *F* is the applied force defined as load [L, kg], *g* = 9.80665 m/s^2^ and *l* is the nozzle length [7.7 mm],

(4) $$\dot{\langle \gamma \rangle }=\frac{4MFI}{600\rho \pi R_{N}^{3}}$$

where *MFI* is the melt flow index (210 °C) [g/10 min], and *ρ* is the density [g/cm^3^].

The above procedure was postulated among others in [39]. In order to estimate the power-law coefficient, the MFI was measured at different loads. Measurements were taken at loads of 1200, 1400, 1700, 2000, 2200, 2400, and 2700 grams. The samples at each load were taken 5 times and errors for MFI, 〈γ〉, τ and η were calculated with the partial derivative of mean squared error (MSE). The obtained consistency index *K* and the power-law exponents were determined from approximation curves to calculate the filament feed rate. Friction test samples were printed as in previous work [29], and their parameters are shown in Table 2.

The tribological properties of the printed composites were determined using a TM-01M tester (pin-on-disc method) in dry friction conditions in reciprocating movement. Tests were carried out for a distance of 120 meters, under a 20 N load, with a velocity of 0.03 m/s, with a cast-iron countersample (φ = 3 mm). Such a friction pair was chosen as a comparative method to assess possible future applications of the composite. Moreover, the impact of the path layout was determined, and friction tests were conducted in three directions: parallel to the outer printed paths (0°), perpendicular to the paths (90°), and at 45°. Tests were performed on three specimens and the presented curves are the representative ones. The average size of wear trace was 4 mm width and 12 mm length. The specific wear rate (SWR) was calculated according to Equation (5):

(5) $$Specific\;wear\;rate\;\left(SWR\right)=\;\frac{\Delta m}{P\cdot L}$$

where Δm is the mass change of a sample, *P* is the load, and *L* is the distance.

Finally, sample surfaces after friction tests were characterized using scanning electron microscope (SEM).

**Figure 3:**
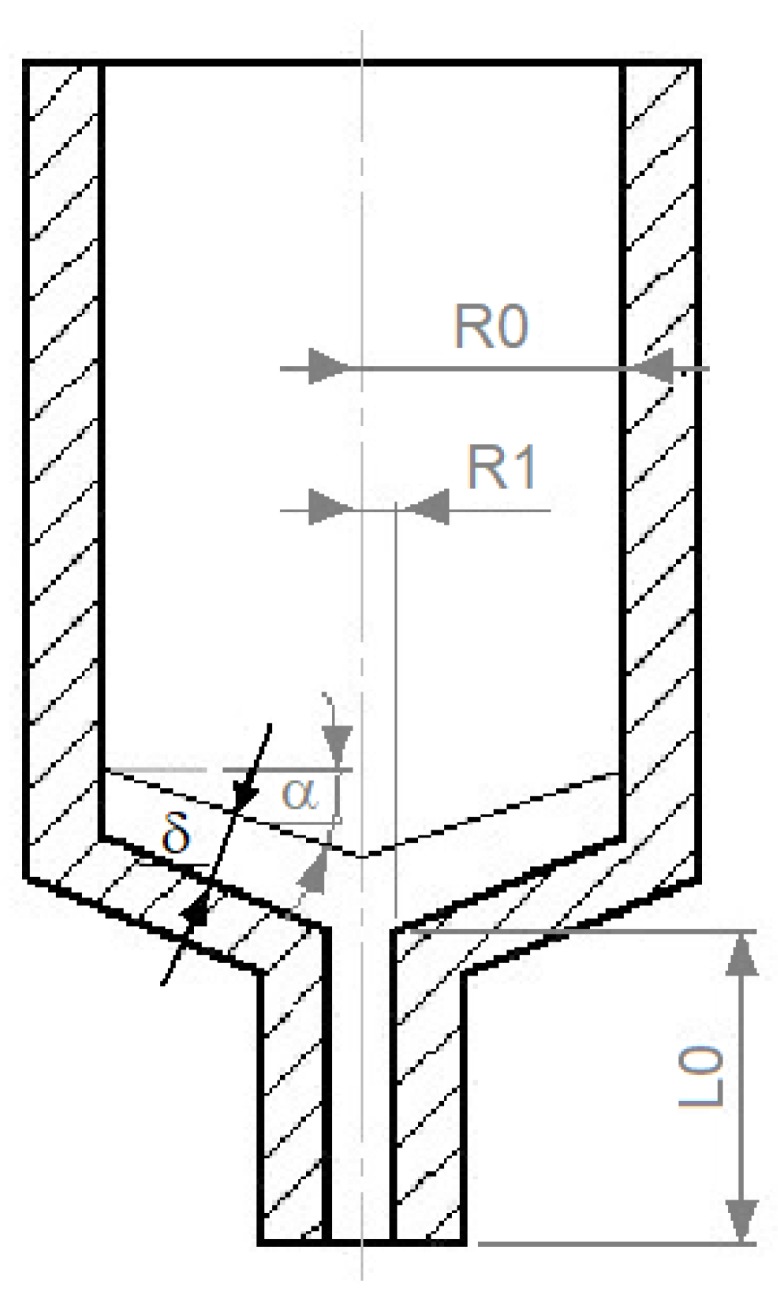
Main dimensions of the melting area of the printer [21].

The maximum filament feed rate was calculated using the mathematical model proposed by Osswald et al. [24] in which a melt filament is pushed towards a conical (or flat in this case) nozzle to form a thin film. The vital dimensions of the melting area of the printer are illustrated in Figure 3.

Such an approach requires melting the material in a liquifier and simultaneously applying a force to the filament, which moves it through the nozzle. In the model, the force applied to the filament can be expressed as a function of the filament feed rate, liquifier geometry, and the material properties (Equations (6) and (7)):

(6) $$\begin{array}{c} F=\left(\frac{6\pi \mu_{f}v\frac{\rho_{s}}{\rho_{m}}R_{0}{}^{4}}{\cos\frac{\beta }{2}\;\delta^{3}}\right)\left[\ln\left(\frac{R_{1}}{R_{0}}\right)+\frac{3}{4}+\frac{1}{4}{\left(\frac{R_{1}}{R_{0}}\right)}^{4}-{\left(\frac{R_{1}}{R_{0}}\right)}^{2}\right]\\ -8\pi \mu_{c}v\frac{\rho_{s}}{\rho_{m}}L_{2}{\left(\frac{R_{1}}{R_{0}}\right)}^{2} \end{array}$$

where *µ*_f_ is the viscosity of the melt film [Pa·s], *ρ*_s_ is the filament density [g/cm^3^], *ρ*_m_ is the melt density [g/cm^3^], *R*_0_ is the filament radius [mm], *R*_1_ is the nozzle radius [mm], δ is the thickness of the melt film [mm], *µ*_c_ is the polymer viscosity in the nozzle [Pa·s], and *L*_2_ is the nozzle height [mm].

(7) $$\delta =\frac{k_{m}\left(T_{d}-T_{m}\right)}{\rho_{s}v\left[\lambda +C_{p}\left(T_{m}-T_{0}\right)\right]}$$

where *k*_m_ is the thermal conductivity of the melt [Wm^−1^K^−1^], *T*_m_ is the polymer melting point [K], λ is the melt enthalpy [J/kg], *C*p is the heat capacity of the filament [Jkg^−1^K^−1^], and *T*_0_ is the initial temperature of the filament [K].

To determine the maximum filament velocity, the least-squares method may be applied. As the boundary condition, a Euler buckling condition (Equation (8)) was used as proposed by Bellini et al. [22]:

(8) $$P_{cr}=\frac{\pi Ed_{0}{}^{2}}{16L_{0}{}^{2}}$$

where *E* is the filament’s Young’s Modulus [GPa], *d*_0_ is the filament diameter [mm], and *L*_0_ is the distance between the rollers and liquifier [mm].

This approach is very suitable because by changing the filament diameter, liquifier geometry, or Young’s Modulus of the material, the critical buckling pressure can be increased. This condition can also be applied for flexible filaments such as LDPE and its composites. The linear printing speed (in either the X or Y direction) was estimated from the calculated feed rate by simply assuming that the printing volume rate equals the fed filament volume.

## 3. Results and Discussion

### 3.1. Composites Characteristics

**Figure 4:**
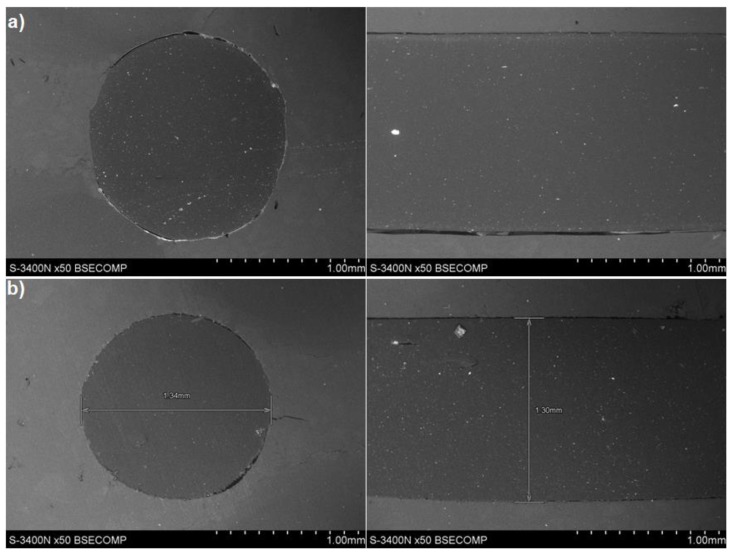
Composite filament microstructure: (**a**) LDPE15, (**b**) LDPE30.

The microstructures of the obtained composites are shown in Figure 4. The reinforcement composite filaments were homogeneously distributed throughout the matrix, and there was also a clearly visible difference in morphology between the composites, representing a different glass powder volume fraction.

**Figure 5:**
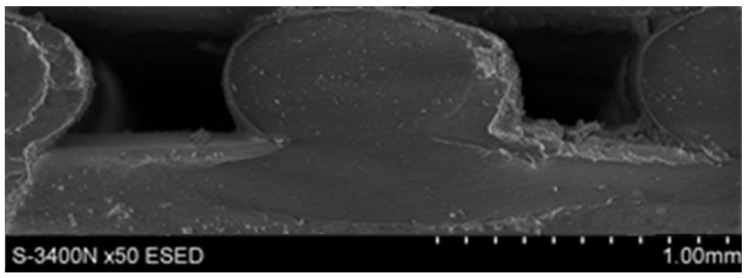
Bonding between path layers in the 3D printout printed from the LDPE30 composite.

After 3D printing (Figure 5), the composites showed good bonding between path layers without cracks or voids, which indicates that the layers were well-melted and mutually permeated.

The MFI values were used to determine rheological coefficients of the composites using the power law (Equation (4)). The MFI indexes determined for the obtained composites at various loads are given in Table 3.

As can be seen in some cases, the introduction of solid particles into a polymer matrix increased its MFI. However, it is only apparent and such effect can be explained with an increased density. The MFI tests are performed in a vertical position, which causes a stronger acceleration from gravity for the materials at higher mass density. In general, the viscosity of composites must be higher than those of neat LDPE, so their flow rate must be lower [40,41,42]. The presented analytical methodology (Equation (4)) uses MFI, but it takes into account the density, so the influence of the "gravitational" factor is neutralized in it. This effect can be confirmed by using MFI to calculate the flow rate Q (Equation (9)) [43]:

(9) $$Q=\frac{MFI}{\rho }$$

For instance, the flow rate of neat LDPE (at load 2000 g) is 3.29 cm^3^/10 min, when for composites that value decrease to 3.14 and 3.03 cm^3^/10 min, respectively for LDPE15 and LDPE30 (Table 4). Similar increases of MFI for LDPE were observed in the work of Bedi et al. [18].

**Figure 6:**
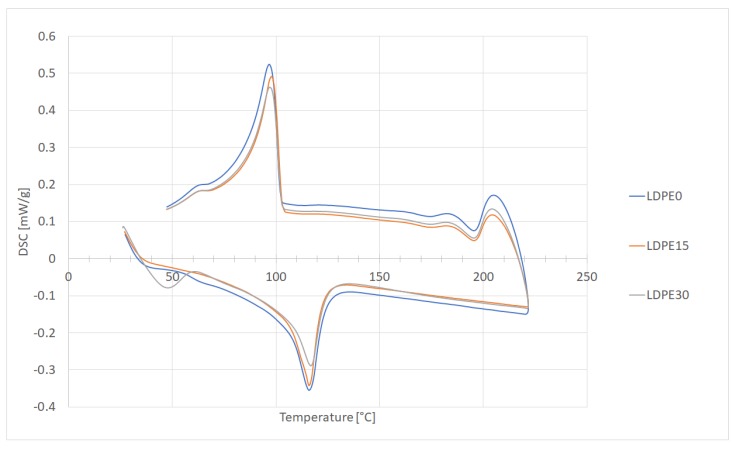
Differential scanning calorimetry (DSC) plots of neat LDPE and the obtained composites.

The melt enthalpy was obtained from DSC analysis (Figure 6). The addition of glass particles did not significantly influence the melting and crystallization temperature. In all samples, a transition at 65 °C was observed, which was related to the relaxation after LDPE crystallization. The composite samples exhibited a higher crystallinity than neat LDPE, which indicates that the glass powder likely influences the nucleation during crystallization. It may also justify above-mentioned changes in the viscosity vs. shear rate relation.

**Figure 7:**
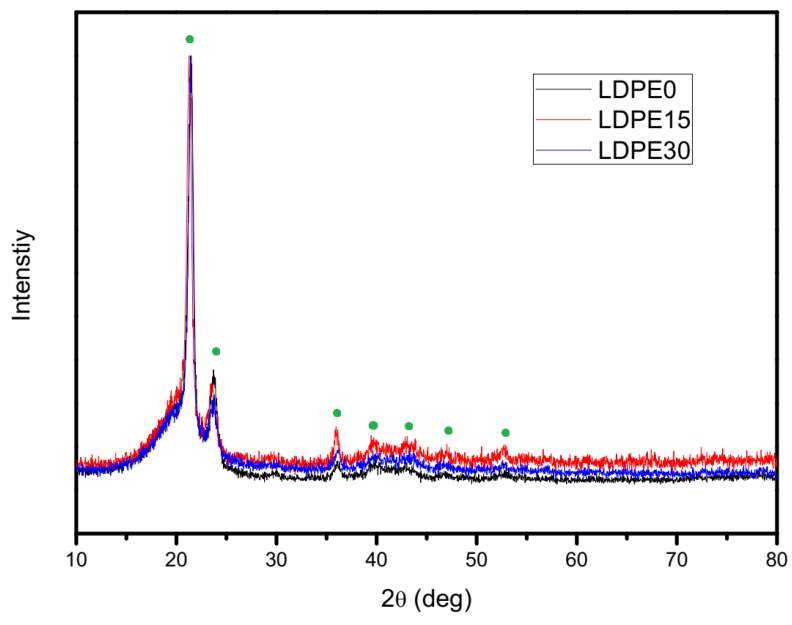
X-ray diffraction plot of neat LDPE and the obtained composites.

Performed XRD analysis confirmed the semi-crystalline structure of neat LDPE and the obtained composites (Figure 7).

The interpretation of the peaks was made according to work by Schmacke [44]. All samples exhibit a similar crystalline form of PE. However, the area of crystal peaks at 21.3° and 23.7° of 2θ (two highest peaks) decreased while the area of the smaller peaks increased. The crystallinity calculated from XRD confirms the DSC measurement, however, the increase in crystallinity for composites is smaller. According to others work [45], fine glass particles (D90 = 20.1 μm) can behave as nucleating agenda similar to talc. To confirm such conclusion additional research in this field is necessary.

### 3.2. Feed Rate Calculation

Most parameters of the model calculation in Table 5 were determined experimentally or measured, but the heat capacity, the thermal conductivity of the melt, and melt density were taken from another source [46].

The values of *C*_p_ and *k*_m_ for LDPE15 and LDPE30 were calculated from a rule-of-mixture equation using the weight fraction (Equation (10)) [31]:

(10) $$A_{c}=A_{m}wt{\%}_{m}+A_{r}wt{\%}_{r}$$

where *A*_c_ is a property of the composite, *A*_m_ is a matrix property, *A*_r_ is a reinforcement property, *wt*%_m_ is the weight fraction of the matrix, and *wt*%_r_ is the weight fraction of the reinforcement. The parameters used to mathematically model the FDM process feed rate (Equations (7) and (8)) for neat LDPE and for the obtained composites are given in Table 6. The melt density of all samples was assumed to be 80% of the solid density, according to [47].

The calculated values of feed rate and printing speed (Table 7) matches the experimentally determined printing parameter in Table 2 (printing speed = 3 mm/s).

**Figure 8:**
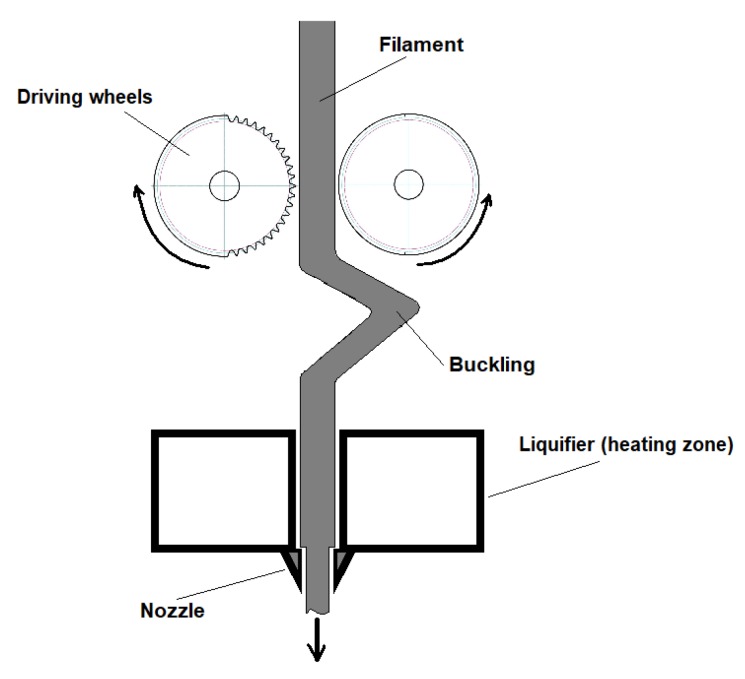
Illustration of filament buckling due to exceeding a printing speed of 3 mm/s.

3D printing these materials at a speed higher than 3 mm/s results in filament buckling directly below the driving wheels (Figure 8).

This indicates that the presented model can predict printing parameters, but there are some differences between the theoretical and experimental values which are the result of inadequately estimating the thermal properties of materials. The modeling results suggest that the composite materials can be fed faster than neat LDPE. Even though the viscosity of composites is higher during printing, the difference is probably compensated for by a higher stiffness along with a change in its thermal properties. In addition, the melt film thickness is higher for LDPE15 and LDPE30, which should lower the tension in the filament. Such a result suggests that in composite filaments, the reinforcement can absorb more heat than the matrix and transfer it to the nozzle, which impacts the melting process. Such behavior should be limited since an increase in the viscosity will have a greater impact on 3D printing than mechanical or thermal changes.

### 3.3. Tribological Properties

**Figure 9:**
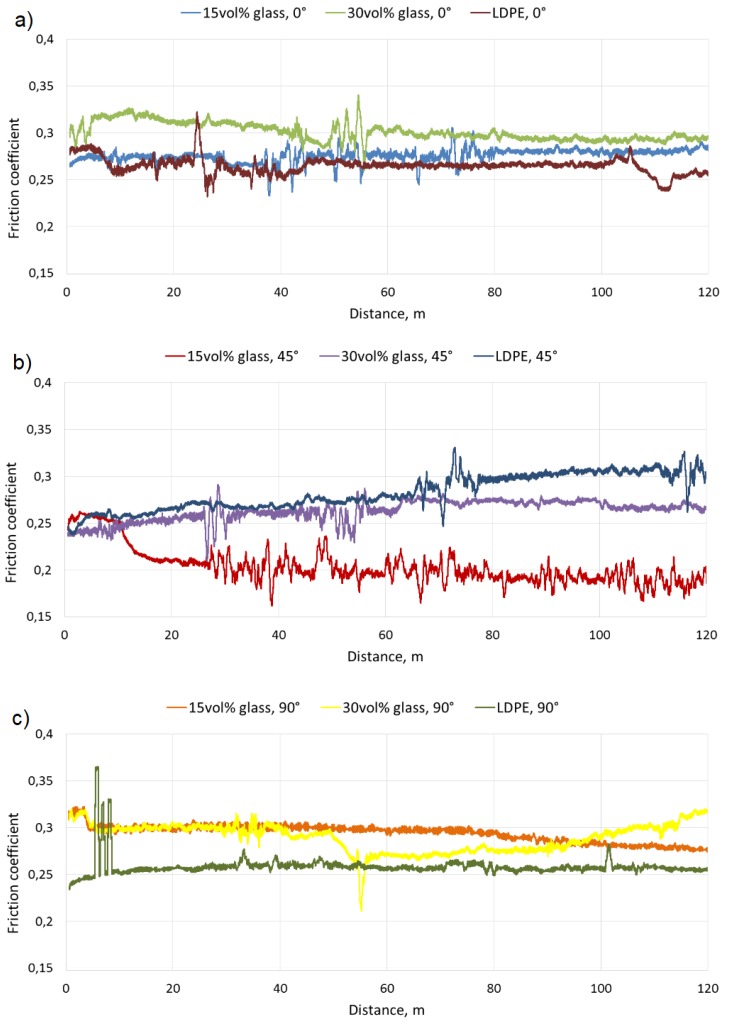
Representative curves of changes of the friction coefficient determined for neat LDPE and for composites over a distance of 120 m, rotated relative to the friction direction by: (**a**) 0°, (**b**) 45°, (**c**) 90°.

**Figure 10:**
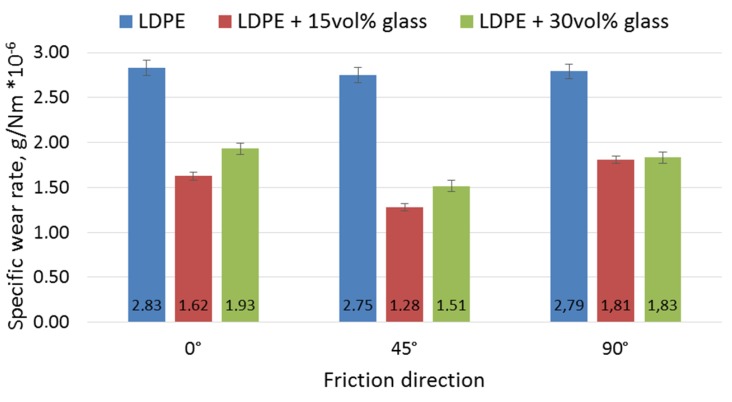
Calculated specific wear rate for examined materials: neat LDPE and the composites.

Representative curves of friction coefficient changes vs. friction distance are presented in Figure 9. The specific wear rate (SWR) of the materials was set in Figure 10.

A slight increase in the average value of the friction coefficient was observed for composites compared with the reference material, but only into 0° and 90° directions. For the 45° direction, the trend is reversed. However, the performed tests showed that the most favorable friction direction for LDPE-based 3D prints was 90°, which was related to the relatively poor bonding between the printed paths. The application of LDPE-based composites with glass particles increases the friction coefficient but also stabilizes it. Unlike the pure polymer, in composites, the most favorable direction was 45°. This direction showed a friction coefficient reduction of approximately 30% for the composite with 15 vol.% glass and approximately 10% for the composite with 30 vol. % glass, compared with the neat LDPE.

**Figure 11:**
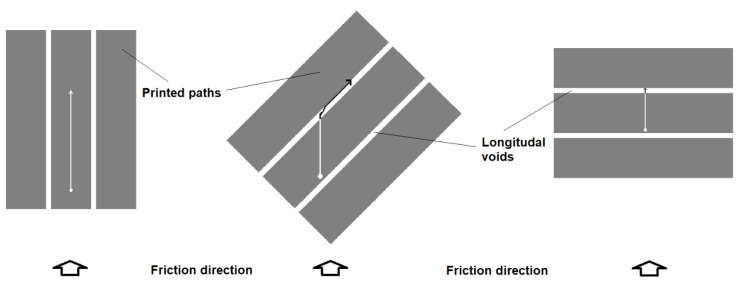
The idea of particle movement after it is separated from the matrix (marked with arrows)—in the three cases of printed paths directions vs. friction direction.

The application of fine glass powder reduced the wear in the examined friction couple in all friction directions. However, the highest SWR reduction (up to approx. 50%) was observed in the 45° printed paths direction, which indicates that this direction was more beneficial when applied to the composites. This is unlike the neat LDPE, for which the SWR was practically independent of the path’s direction. The effect of the path’s direction was even stronger in the composite with 15 vol. % of glass powder than for the 30% one, suggesting some complex friction mechanisms in this case. Most likely, hard glass particles skidded from the friction area and created a kind of film on the surface which facilitated countersample movement. These particles acted as microscopic rolling bearings in a mechanism described in [48] and [19]. In order for this film to be effective, the number of particles per unit area must be adequate—neither too small nor too large—and this number undoubtedly depends on the volume fraction of particles in the composite. To precisely define it, a separate test procedure would have to be carried out, but it can be concluded from the results shown in Figure 10 that the 15% composite was more optimal than the 30% one. The number of particles in the film also depends on the intensity of the removal of particles from the friction area, which depends on the direction of the printed tracks in the case of a 3D-printed element. The track of the potential particle skidded by the countersample, at different track directions, is shown in Figure 11.

The track is limited by the longitudinal voids between the paths (see also Figure 5). Most likely, for the optimal effect, the film must achieve "stability", which it does achieve when the distance tracked by the skidded particles is too short (90° position) or too long (0° position). The most optimal direction seems to be 45°. The path of the skidded particles is long enough that the right number of them creates a film, but short enough that there are not too many particles, which results in "seizing" of the system and intense wear. Similar mechanisms for metal composites were precisely analyzed, among others in [48]. The obtained results do not indicate that the texture of the material caused by the printing process significantly influences its wear resistance.

**Figure 12:**
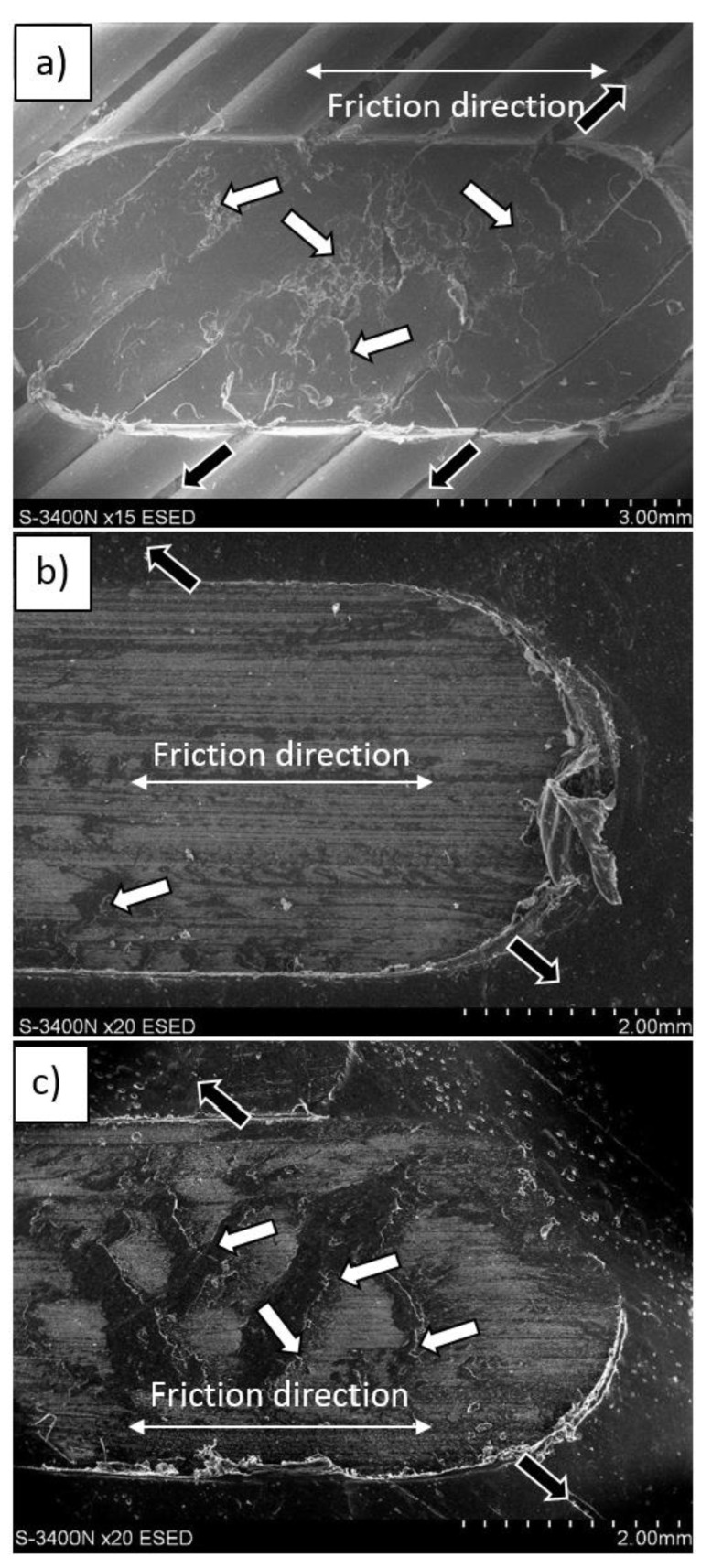
SEM micrographs of surfaces after friction tests: (**a**) LDPE, (**b**) LDPE-15% vol. glass, and (**c**) LDPE 30% vol. glass. The test was performed at a 45° angle, and white arrows indicate areas of plastic deformation and delamination, while black arrows indicate the direction ("canals") of friction products removal.

**Figure 13:**
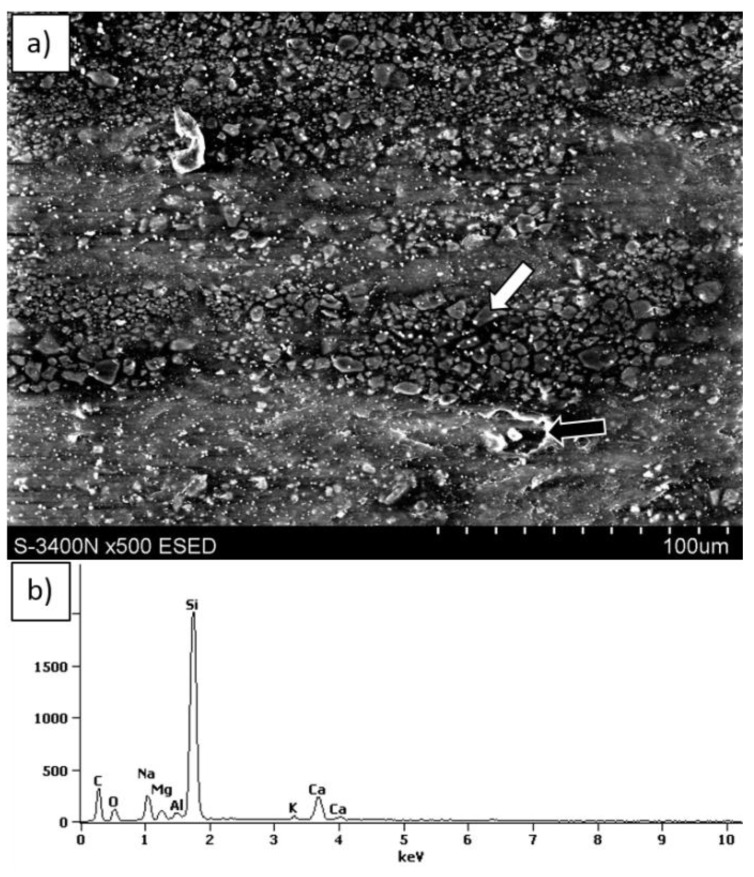
(**a**) Representative SEM micrograph of the LDPE-30% vol. glass composite surface after conducting a friction test with the corresponding energy dispersive spectroscopy (EDS) spectra (**b**) of stacked particles (white arrow). The black arrow indicates a microregion of plastic deformation.

SEM observation of sample surfaces after tribological test showed that glass particles separate from the matrix. In the next step, the wrested particles were stuck again in the LDPE matrix (Figure 12 and Figure 13). This effect is more intense when the composite with 15% vol. glass powder was applied (Figure 12, bright areas).

The dark areas visible in the SEM micrographs represent the plastic deformation of LDPE. It should be noted that adding 15 vol.% reinforcing powder was more beneficial due to the better arrangement of glass sticking, since there were larger areas of neat LDPE between the particles, compared with the 30%. Accumulation of friction products was observed in the side region of the wear trace in the 30% composite (Figure 13). Energy dispersive spectroscopy (EDS) tests showed that the products (building the tribological film) consisted solely of glass, which justifies the different wear behavior of the composites described above.

Basing of presented feed rate calculation model there is possible to predict a behavior of composites with different glass particles volume fraction (e.g., 50%). Collected data about investigated materials (LDPE0, LDPE15, and LDPE30) showed that the increase of the reinforcing phase would cause a slight increase in melt viscosity, would not have a significant effect on the crystallinity of the matrix and—due to the above-described friction mechanisms—probably would not improve the wear resistance of the material.

The results obtained in the study are of great practical importance. They show, among others, that:

- you can produce low-duty friction material from waste—waste glass was used in the work, but the LD-PE used as a matrix also can be a waste material, probably with the highest tonnage of all materials.
- the frictional properties of the soft (and cheap) polymer can be significantly improved by adding a relatively cheap reinforcing component.
- you can optimize the path layout in the FDM printed material so as to minimize frictional wear.
- simple and easily determinable coefficients (e.g., MFI) can be used to predict the technological properties of FDM-related materials, which can greatly simplify and speed up the procedure for implementing new printing materials.

## 4. Conclusions 

In this preliminary work, the technological properties (filament feed rate and printing speed) and wear resistance (specific wear rate) of novel 3D-FDM printed LDPE-matrix composites were evaluated. The following general conclusions were drawn:

The applied procedure using a mathematical model was used to estimate the maximum feed rate and hence the printing speed. Such use is effective and helpful for researching new 3D printing materials.

The composites showed a higher elastic modulus compared with neat LDPE. An improvement in the elastic modulus allows a faster print speed, but failure in printed parts may be observed due to faster printing.

Composite samples exhibited a higher crystallinity than neat LDPE. However, the increase is very slight and close to the error limit.

Using a fine glass powder as a reinforcement in the thermoplastic polymer composites improved their wear resistance due to the formation of a sliding film on the sample surface.

An evident effect of the friction direction vs. the printed path direction on the wear appeared, which was probably related to differences in the removal of friction products from the friction area for different print-path layouts against the friction direction.

The LDPE composite with auto-screen glass particles is a promising material and should be studied further.

## Figures and Tables

**Table 1:** Young’s modulus of the composites.

	LDPE0	LDPE15	LDPE30
**Glass content, vol %**	0	15	30
**Young’s Modulus [GPa]**	0.193 (0.010)	0.208 (0.016)	0.219 (0.007)

**Table 2:** Established printing parameters.

Nozzle Diameter	Build Plate Temperature	Printing Temperature	Layer Thickness	Printing Speed	Printing Speed of the First Layer	Flow Rate	Cooling
1 mm	70 °C	210 °C	0.5 mm	3 mm/s	50%	80%	none

**Table 3:** **Mold flow index (**MFI) results for the neat LDPE and for the obtained composites.

**LDPE**							
**Load [g]**	**1200**	**1400**	**1700**	**2000**		**2200**	**2400**
MFI [g/10 min]	1.35 (0.07)	1.63 (0.08)	2.19 (0.11)		2.97 (0.15)	3.35 (0.17)	3.6 (0.18)
**LDPE15**							
**Load [g]**	**1200**	**1400**	**1700**		**2000**	**2200**	**2450**
MFI [g/10 min]	1.30 (0.04)	1.61 (0.05)	2.18 (0.07)		2.96 (0.10)	3.42 (0.11)	4.25 (0.14)
**LDPE30**							
**Load [g]**	**1200**	**1450**	**1700**		**2000**	**2200**	**2450**
MFI [g/10 min]	1.70 (0.11)	1.93 (0.13)	2.22 (0.07)		2.98 (0.10)	3.55 (0.14)	4.28 (0.14)

**Table 4:** Flow rate (Q) values obtained for tested composite melts.

Parameter	LDPE0	LDPE15	LDPE30
Flow rate Q [cm^3^/10 min]	3.29	3.14	3.03

**Table 5:** Power law coefficients (Equations (1) and (7)), melt enthalpy, crystallization enthalpy, and crystallinity of the neat LDPE and the composites.

	K [Pas]	n	T_m_ [K]	T_c_ [K]	ΔH_m_ [J/g]	ΔH_c_ [J/g]	χ_m_%	χ_c_%
**LDPE0**	4066	0.647	115.96	96.87	56.66	57.89	19.3	19.8
**LDPE15**	4563	0.601	115.97	98.1	56.13	55.44	23.6	23.5
**LDPE30**	4752	0.595	117.24	96.84	58.74	53.47	27.4	25

**Table 6:** Parameters used in mathematical modeling of FDM process feed rate for the neat LDPE and for the obtained composites.

Parameter	LDPE0	LDPE15	LDPE30
Solid density [kg/m^3^]	902.04	942.70	983.45
Melt density [kg/m^3^]	721.63	754.16	786.76
Young Modulus [GPa]	0.200	0.208	0.219
k_m_ [W/m K]	0.33	0.45	0.49
C_p_ [J/kg K]	2300	2028	1899
Printing temperature [K]	483	483	483
Nozzle length [mm]	1	1	1
Nozzle angle [°]	55	55	55
Nozzle diameter [mm]	1	1	1
Distance between rollers and liquifier, L_f_ [mm]	45	45	45
Critical pressure, P_cr_ [kPa]	140.9	146.5	154.3

**Table 7:** Results of mathematical modeling: melt thickness, feed rate, and printing speed (Equations (7) and (8)).

Property	LDPE0	LDPE15	LDPE30
Melt thickness [µm]	279.91	373.44	379.86
Feed rate [mm/s]	0.72	0.83	0.89
Printing speed [mm/s]	2.38	2.75	2.95
